# Enhancing Primary Care for Nursing Home Patients with an Artificial Intelligence-Aided Rational Drug Use Web Assistant

**DOI:** 10.3390/jcm12206549

**Published:** 2023-10-16

**Authors:** Tuğba Yılmaz, Şükran Ceyhan, Şeyma Handan Akyön, Tarık Eren Yılmaz

**Affiliations:** 1 Department of Family Medicine, Ankara Bilkent City Hospital, Ankara 06800, Türkiye; sukran.kanoglu@saglik.gov.tr (Ş.C.); seymahandan.dogan@saglik.gov.tr (Ş.H.A.); 2 Department of Family Medicine, University of Health Sciences, Gülhane Training and Research Hospital, Ankara 06010, Türkiye; tarikeren.yilmaz@sbu.edu.tr

**Keywords:** polypharmacy, rational drug use, geriatrics, primary care, medical informatics applications, quaternary prevention, cost-effectiveness analysis

## Abstract

Polypharmacy can result in drug–drug interactions, severe side-effects, drug–disease interactions, inappropriate medication use in the elderly, and escalating costs. This study aims to evaluate nursing home residents’ medication regimens using a rational drug use web assistant developed by researchers to mitigate unnecessary medication usage. This analytical, cross-sectional study included data from nursing home residents recently recorded in a training family health center. Sociodemographic information, medical conditions, and prescribed medications of all patients in the nursing home (*n* = 99) were documented. Medications were assessed using an artificial intelligence-aided rational drug use web assistant. Instances of inappropriate drug use and calculations of contraindicated drug costs were also recorded. The study revealed that 88.9% (*n* = 88) of patients experienced polypharmacy, with a mean value of 6.96 ± 2.94 drugs per patient. Potential risky drug–drug interactions were present in 89.9% (*n* = 89) of patients, contraindicated drug–drug interactions in 20.2% (*n* = 20), and potentially inappropriate drug use in 86.9% (*n* = 86). Plans to discontinue 83 medications were estimated to reduce total direct medication costs by 9.1% per month. After the assessment with the rational drug use web assistant, the number of drugs that patients needed to use and polypharmacy decreased significantly. This study concludes that the rational drug use web assistant application, which is more cost-effective than the traditional manual method, assisted by artificial intelligence, and integrated into healthcare services, may offer substantial benefits to family physicians and their geriatric patients.

## 1. Introduction

Individuals may have several diseases and have to use a lot of drugs for therapeutic purposes for these diseases. There may be drug–drug interactions when more than one drug is used. In this case, patients can be exposed to several side-effects instead of being treated. Regression in organ functions depending on pharmacokinetic and pharmacodynamic changes occurs due to aging, and for this reason, elderly patients are more exposed to the side-effects of drugs used for treatment [1]. Therefore, it is necessary to be sensitive in terms of drug side-effects in elderly patients, and drug side-effects should be taken into account for any new symptoms [2]. Inappropriate drug use has a great impact on the risk of drug side-effects and a decrease in cognitive functions [3]. In this manner, appropriate drug use is crucial, particularly in elderly patients [4].

The use of some drugs is inappropriate for patients above the age of 65. The term “polypharmacy” is generally known as the use of five or more drugs at the same time. However, as well as the number of drugs, inappropriate indications and the use of two or more unnecessary drugs together are also known as polypharmacy [5,6,7].

Polypharmacy may be caused by the patient or the healthcare system. Regarding patient-related conditions, the number of drugs used for chronic diseases increases, and therefore, polydrug use appears [8]. However, concomitant diseases in individuals independent of age also cause polydrug use and thereby the increase in related side-effects [1].

Conditions such as an insufficient number of family physicians who will coordinate drug treatment, extremely busy schedules of physicians, physicians’ lack of knowledge on side-effects of the drug, and drug–drug interactions can be among the factors regarding the healthcare system [8]. Moreover, “prescribing cascade”, which is defined as prescribing another drug to treat the side-effect of a drug, is also another important cause of polypharmacy [9,10].

Drug–drug interaction, side-effects of drugs, drug–disease interaction, inappropriate drug use in the elderly, and increase in treatment costs, hospitalization, and mortality are among the complications of polypharmacy [7]. As the number of drugs used increases, side-effects of drugs increase.

Family physicians provide primary healthcare services and several patients visit them to have their drugs prescribed. While prescribing the drugs of patients here, assessment of drugs, diseases, drug–drug interactions, drug appropriateness, and rational drug use become crucial. Moreover, family physicians approach patients in a holistic way and evaluate them with all their diseases and keep their patients from overmedicalization through quaternary prevention. In this regard, they have crucial responsibilities in regulating patients’ drugs.

Our study aimed to assess patients staying in a nursing home in terms of diseases, drugs used, drug–drug interactions of these drugs, and appropriate use of these drugs by age through a rational drug use web assistant [7] developed by researchers. Therefore, the drugs of these patients were checked, and inappropriate or unnecessary drugs were detected; cessation of these drugs after consulting related departments could be possible. Decreasing the rates of mortality, morbidity, hospitalization, and side-effects and the direct and indirect unnecessary costs of these were targeted by preventing patients from using unnecessary and unregulated drugs.

## 2. Materials and Methods

Our research is an observational and cross-sectional study. A web-assisted application for rational drug use [7] that was developed by the researchers and used in the healthcare center in which we provided primary healthcare services was utilized in our study. In addition, a data collection form structured particularly to the study established with parameters that were determined by the researchers was used.

The study population consisted of all patients staying in the nursing home who had recently been recorded at the Çayyolu Training Family Health Center of Ankara Bilkent City Hospital. All volunteer patients from the nursing home (*n* = 99), whose records were available in our healthcare center, were included in the study between 30 July and 30 August 2023.

### 2.1. Data Collection Tools

In our study, patients’ age, gender, occupation, educational status, marital status, diseases, drugs used were collected. Patients’ information and drugs used were obtained from the patients themselves or from those who constantly gave primary care to the patients and were scanned and controlled from the related e-Pulse database, the national health database. E-Pulse is a personal health record system integrated by the Turkish Ministry of Health into all healthcare institutions’ information systems. This innovative platform enables individuals to access a comprehensive range of medical information, including laboratory results, medical images, prescription and medication details, emergency information, diagnosis details, reports, and comprehensive health records, all of which pertain to their examinations. Accessible through both desktop and mobile platforms, e-Pulse empowers individuals to manage their healthcare effectively. Additionally, the platform allows users to securely share their medical records with healthcare professionals and family members, ensuring confidentiality within specified regulations [11]. Drug–drug interactions of these drugs and their appropriateness to the disease or age were assessed via a rational drug use web assistant [7] developed by the researchers. Reports of the web assistant were also evaluated by physicians in this study. Cancellation of PPI therapy in the prescription which was in the control of primary healthcare physicians was regulated by the physicians designing the study. However, regulation of psychiatric drugs such as quetiapine was performed by consulting the related department.

### 2.2. Rational Drug Use Web Assistant and Its Features

Before this study, an artificial intelligence-aided web application with rule-based algorithms was developed to simplify patient assessment based on age, medications, and chronic diseases in clinical practice [7]. This artificial intelligence system uses an extensive database to quickly retrieve interaction information and makes decisions based on predefined rules from pharmaceutical sources and the medical literature. It also offers access to the sources for decision justification.

In the database of the artificial intelligence-aided *Fast&Rational* rational drug use assistant (**http://fastrational.com/** accessed on 30 July 2023), 6 guides (*TIME-to-STOPP, AGS Beers 2019, US-FORTA, Stopp Criteria v2, EU (7) PIM, and PRISCUS 1.0 List*) revealing the criteria of Potentially Inappropriate Medication (**PIM**) use in the elderly which are commonly used in today’s literature and health service presentation are used.

Before this web application, 430 active and frequently used oral drug agents (parenteral forms, suspended drugs, foreign drugs, and canceled drugs are not included) in Turkey and the 70 most common chronic diseases/medical conditions were identified. Three separate sheets were made with the Microsoft Excel 2021 program. Information on whether 430 drug agents are inappropriate in patients over the age of 65 was recorded in the first sheet, drug interactions were recorded in the second sheet, and drug–disease interaction tables were made in the last sheet. First, these 430 drug agents were screened with 6 PIM criteria. Additional dose information and duration of drug use in the criteria related to each drug agent were added as comments. Information about the source from which each output and comment was taken was also added. In the second stage, a table of interactions of these 430 drug agents was made and interaction information was scanned from drug prospectuses, pharmacology medical books, and related articles published in peer-reviewed journals. In the third stage, a drug–disease interaction table was made, and interaction information was searched from drug prospectuses, chronic disease guides, pharmacology medicine books, and articles published in peer-reviewed journals. According to these sources, interaction information was classified into 3 different categories as “risky (orange color)”, “contraindicated (red color)”, and “no warning was found”. Additional information about the interaction was added to the table along with the sources from which it was taken from as comments. Then, the algorithms of the tables which were prepared in Microsoft Excel 2021 (drug–age, drug–drug, drug–disease) were made by an expert artificial intelligence engineer using a rule-based artificial intelligence supported system. The web application interface was made with these algorithms. The web application’s interface design was developed with the Python version 3.10 programming language using the Streamlit Library.

Moreover, this web application is actively used in our clinic.

### 2.3. Cost Analysis

First of all, a cost-effectiveness analysis was performed between the manual approach, described as the traditional and gold standard method, in which the related guide, prospectus, and medical information based on current evidence were assessed and concluded by the physician, and our artificial intelligence-aided rational drug use web assistant including all related guides and prospectus information. Nursing home patients’ initial conditions managed by the traditional method before web assistant use were accepted as the basal level during the related polypharmacy process, and the state of “doing nothing”, in other words, no additional interference, used in cost-effectiveness analyses was defined as the alternative method [12] and considered in our study.

**Figure 1:**
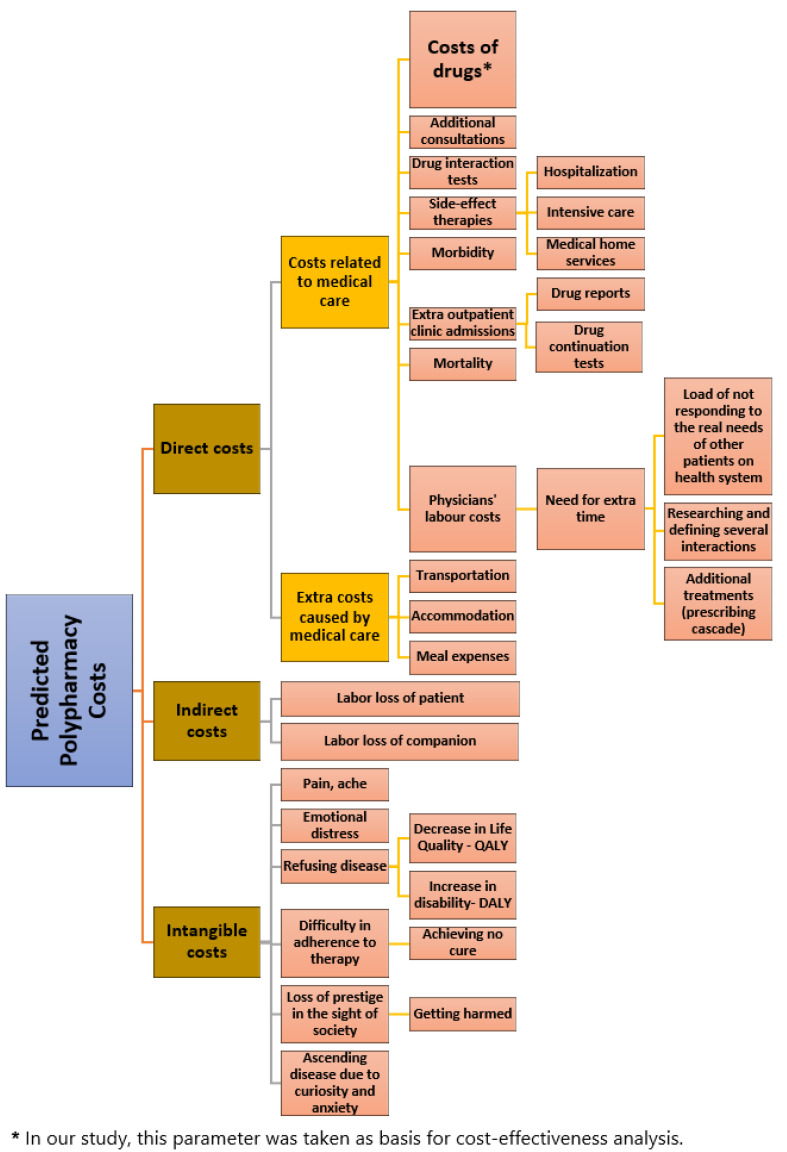
Potential costs of polypharmacy.

Potential polypharmacy costs were primarily collected by scanning the related literature [13,14,15], described and summarized (Figure 1) before the analysis of cost-effectiveness within the scope of this study.

Cost-effectiveness analysis was performed between the two methods (traditional method and web assistant method) in terms of the costs of drugs stated as the leading cause of costs related to medical care, regarding only direct costs among several potential cost items of polypharmacy. Therefore, all of the patients’ drugs obtained from the related patient files and their caregivers and confirmed in the e-Pulse national database were documented in Excel format. Prices of the related drugs were noted down and listed by specialists one by one according to the prices defined as the public price in Turkey and determined on a legal basis as cheaper than the retail price in the repayment cover. Because only the generic names of drugs were anonymized and given to the individuals who would note the related drug fees down and due to the abundance of drugs for patients, the drugs were conveyed regardless of their usage periods or doses. A drug was selected among those drugs whose generic names were given, and the average dose was based among all the doses of the drug selected as the most affordable one among all its equivalents in the market. It was considered that, for each drug, only one pack was used in a month and the total cost was obtained by calculating the minimum average costs. Thus, total costs were compared for the initial states of patients and the states as a result of drug regulation after web assistant use. Cost calculation was performed in currency, and effectiveness was measured with the frequency of exposure to polypharmacy, a quantitative medical outcome in our study, which is suitable for a general approach in these kinds of cost-effectiveness analyses.

### 2.4. Statistical Analysis

Statistical analysis of all data was performed with the SPSS 25.0 software program after combining it in a common database. For descriptive statistics, continuous variables were expressed in mean ± standard deviation and discrete data in numbers and percentile. Whether continuous variables were normally distributed or not and histogram graphs were evaluated with interpretations of Kolmogorov–Smirnov and Shapiro–Wilk tests. One sample *t* test was used to find out whether the mean value of a variable exhibited a difference according to a determined constant number. Correlation analysis was performed to determine the correlation between two or more variables and detect the power and direction of the correlation if available. The chi-square test was used for intergroup and intragroup comparisons of qualitative data. The McNemar test was used to analyze paired nominal data related to the polypharmacy status of nursing home patients before and after the assessment conducted through the web application. Confidence interval was determined as 95% for differences between groups, and *p* < 0.05 was accepted as the significant value.

## 3. Results

A total of 99 individuals staying in a nursing home were included in our study. The mean age of the patients was 79.81 ± 7.64 (min: 62, max: 99). Of the patients, 63.6% were female and 47.5% were widowed or divorced. Sociodemographic information of the patients is given in Table 1.

In our study, 18.2% (*n* = 18) of the total number of patients were using fewer than 5 drugs, 69.7% (*n* = 69) were using 5–10 drugs, and 12.1% (*n* = 12) were using 11–14 drugs. Inappropriate drug use was detected in 81 individuals using five or more drugs and in 7 individuals using fewer than five drugs, which means 88.9% (*n* = 88) of the patients were exposed to polypharmacy.

The mean number of drugs used by the patients in our study was 6.96 ± 2.94. The highest number of drugs used was 14, and there was only one patient who had not been using any drugs. In addition, the number of patients’ diseases had been assessed and the mean number of diseases was calculated as 6.37 ± 2.97 (minimum: 0; maximum: 13).

In our study, drug–drug interaction and potentially inappropriate drug use above the age of 65 in our nursing home patients who had recently been recorded were determined with a rational drug use web assistant established by the researchers and used within the context of preventive healthcare services in our primary healthcare center.

### 3.1. Detailed Classification of Drug–Drug Interaction and Contraindicated Drugs

As a result of the assessment of drug–drug interaction with a rational drug use web assistant, potential risky drug–drug interaction was detected in 89.9% (*n* = 89) of the patients. Contraindicated drug–drug interaction was detected in 20.2% (*n* = 20) of the patients. When contraindicated drug interactions were assessed, an interaction with a minimum of two and a maximum of four active agents was detected. The number of patients who had contraindicated drug–drug interaction with two active agents was 14, and 71.4% (*n* = 10) of them had quetiapine drug interactions. Quetiapine drug interaction was monitored in one half and rasagiline drug interaction was monitored in the other half of the patients (*n* = 4) who had contraindicated drug–drug interaction with three active agents. In those who had contraindicated drug–drug interaction with four active agents (*n* = 2), quetiapine, rasagiline, and olanzapine drug interactions were observed.

### 3.2. Potentially Inappropriate Drug Use above the Age of 65

Potentially inappropriate drug use was detected in 86.9% (*n* = 86) of geriatric patients. Proton Pump Inhibitor (PPI) use was the most common with a rate of 64% (*n* = 55) among these patients. Potentially inappropriate drug use of quetiapine was reported in 31.4% (*n* = 27) of the patients.

According to the recommendations of the web assistant and the evaluation of our physicians, it was determined that a minimum of one and a maximum of three active agents must be removed from the drugs used in 58 (58.6%) patients. The related drugs were ceased in cooperation with the family physicians conducting the study and the physicians of the related departments they consulted. In this way, all contraindicated drug–drug interactions with the drugs planned to be taken out (*p* < 0.001) were removed, and potentially inappropriate drug use above the age of 65 was significantly decreased (*p* < 0.001) (Table 2). As a result, 12.2% (*n* = 83) of total active agents (*n =* 683) were able to be removed from the drug lists. On the other hand, the related web assistant recommended 13 patients to add PPI into their treatment plans. The recommendations were assessed by the physicians in this study and considered appropriate, and PPI was added to the treatment.

No significant difference was observed between the sociodemographic findings of patients (age, gender, marital status, educational status, and occupation) and the presence of drug–drug interactions and inappropriate drug use above the age of 65 (*p* > 0.05).

A significant difference was detected between the number of drugs used by patients before and after the assessment with the rational drug use web assistant and is given in Table 2.

The correlation of the number of diseases patients had and the number of drugs they used with the number of drugs before and after the assessment of the web application and costs of ceased drugs is given in Table 3.

### 3.3. Results of Cost-Effectiveness Analysis

**Figure 2:**
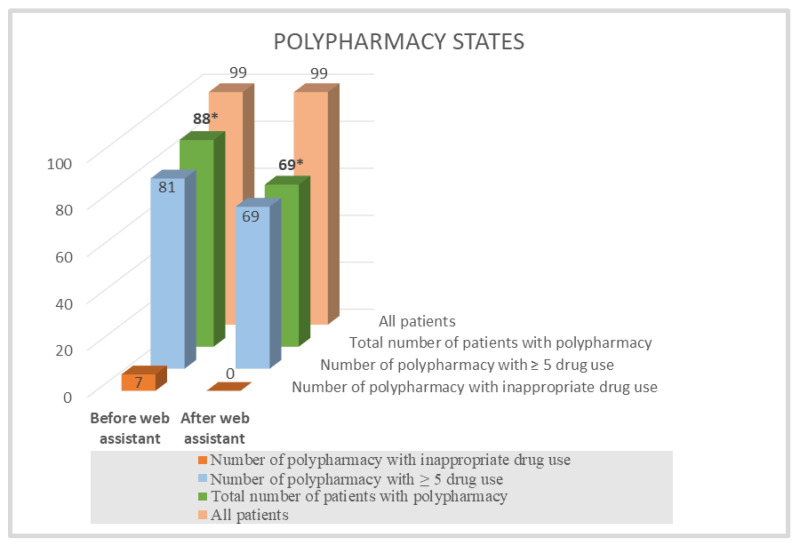
Distributions of polypharmacy states before and after web assistant use in cost-effectiveness analysis. * A significant decrease was observed between polypharmacy states of nursing home patients before and after rational drug use web assistant use (*p* < 0.001).

The total medication cost of drugs used by the nursing home patients was calculated to be 115,232 Turkish Liras (TRY) (about USD 17,071 based on the real price and the currency, determined using legal regulations for the drugs) according to the total number of active agents (per pillbox and at the prevailing Social Security Institute public price). The direct cost of drugs planned to be ceased was calculated to be TRY 10,434 (USD 1545) in total (per pillbox and over public price), which accounts for 9.1% of all drugs used by all patients included in our study. On one hand, this extra cost stood for TRY 105 (USD 15) on average per patient. On the other hand, PPI addition was planned for 13 patients as a result of the web application’s suggestions, and the total cost was calculated to be TRY 825 (USD 122). The total cost obtained and the total cost as a result of the removal of the drugs that was predicted to decrease by the recommendation of the web assistant and physician’s decision were compared for the cost-effectiveness analysis. It was accordingly determined that drug costs decreased by a rate of 9.1% (*p* < 0.001) (Table 2), polypharmacy decreased by a rate of 19.2% in the total population, and the costs decreased by a rate of 21.6% in those with polypharmacy (*p* < 0.001) (Figure 2).

## 4. Discussion

The increase in average lifespan and thereby the elderly population, the number of chronic diseases, and reasons such as natural disasters have recently increased the workload of a clinician and decreased the time to be spent on the patient [16]. This raises the importance and place of artificial intelligence and web assistant implementations in health in order to facilitate the jobs of clinicians [17]. Artificial intelligence implementations help the clinician in the processes of diagnosing diseases, predicting the prognoses of diseases, and deciding on the treatment [17]. These implementations are expected to help physicians in diagnosis, treatment, and follow-up processes rather than replace them [18,19]. There are some artificial intelligence implementations revealing drug–drug interactions [20,21,22]. There are also online websites facilitating physicians to prescribe elderly patients [23,24,25,26,27]. In our study, the drug use of nursing home patients recently recorded in a training family health center was assessed with a rational drug use web assistant we designed with the largest database in the literature. The presence of polypharmacy and risky drug–drug interactions were detected in a high majority of patients in our study with the help of this web application.

Most of the patients in our study had a drug use history with 5–10 drugs. In a study performed by Ozbek et al. to investigate adherence to treatment in 56 elderly patients staying in a nursing home, more than half of the patients were using five or more drugs [28]. According to a study performed by Wawruch et al. [29] on 600 patients at the age of 65 and above in Slovakia, the rate of polypharmacy was 60.3%. In a study performed by Onder et al. [30] on 4023 elderly patients staying in nursing homes in eight different European countries, the rate of polypharmacy was 74%. According to the findings in our study and the literature, it is obvious that polypharmacy is common among the elderly staying in nursing homes. When regulating a new treatment plan for these patients, it should be considered that elderly patients are in the vulnerable patient group and have several chronic diseases. It is also important to obtain information about the drugs they use. Regulating the treatment and nursing care plan [31] is of utmost importance for patients who face increased complexity due to polydrug use. In this regard, we can say that a rational drug use web application is an undeniable necessity. It is considered that not only will the patients be protected from several potential risks particularly caused by the side-effects of drugs but also the unnecessary use of the healthcare system will be prevented with this web application by easily controlling drugs and ensuring appropriate drug use.

The mean number of drugs used by the patients in our study was 6.96 ± 2.94. In a thesis study [32] assessing inappropriate drug use in 1263 elderly individuals, the mean number of drugs used by the patients was 6.10 ± 3.40. The mean number of drugs used by the patients was 5.4 ± 3.24 in a study performed on the elderly by Kitis et al. and 7 ± 3.60 in the study by Lee et al. [33,34]. Regarding the findings in our study and the literature, the mean number of drugs used by elderly patients indicates polypharmacy. Polypharmacy appears a crucial health problem in elderly patients. Controlling the drugs used by these patients and preventing unnecessary drug use are of great importance. Increasing the number of drugs used can increase the number of side-effects as well, and prescribing cascade may occur in geriatric patients. As this may cause significant health problems for elderly patients, the utilization of web applications for rational drug use seems important for the prevention of PIM.

When drug–drug interaction was evaluated with the rational drug use web assistant, potential risky drug–drug interaction was detected in the majority of patients and contraindicated drug–drug interaction was detected in about one-fifth of the patients. According to the assessments of contraindicated drug interactions in our study, drug interaction with a minimum of two and a maximum of four active agents was detected, and contraindicated drug–drug interactions with two active agents were mostly due to quetiapine. Quetiapine drug interaction was observed in one half and rasagiline drug interaction was observed in the other half of the patients who had contraindicated drug–drug interactions with three active agents. In those who had contraindicated drug–drug interaction with four active agents, quetiapine, rasagiline, and olanzapine drug interactions were observed. The most common disease in our nursing home population was hypertension followed by neuro-psychiatric disorders such as depression, dementia, and anxiety, which confirms that. In a study on elderly patients, it was revealed that the use of some drugs such as digoxin, beta blockers, diuretics, antidiabetics, anticoagulants, nonsteroidal analgesics, and psychiatric drugs increased particularly the side-effects and interactions [35]. In the literature, antidepressants and antipsychotics have been revealed to create high risks for drug interactions [36,37,38]. It has also been stated in the literature that antiepileptic and anticholinergic drugs, drugs suppressing the central nervous system, and acetylcholinesterase inhibitors create high risks for elderly patients in terms of drug interactions [39,40,41,42]. Findings in our study and the literature reveal that it is crucial to be careful, especially about the use of drugs affecting the central nervous system in elderly patients. The number of chronic diseases may be high in elderly patients, which may require the use of more than one drug. In this regard, it is especially crucial for family physicians who can approach their patients in a holistic way to follow up on their patients. It is possible to say that the web application we used in our study will provide great convenience for family physicians holistically approaching their patients in the assessment of drugs used.

When potentially inappropriate drug use above the age of 65 was assessed in our study, potentially inappropriate drug use was detected in most of the patients. PPI use was the most common among patients and quetiapine use was detected as potentially inappropriate drug use in about one-third of patients. In studies performed on the elderly in the literature, it is observed that PPIs are prescribed as off-label [43,44]. In a study performed by Çelikci on elderly patients in palliative care service, potentially inappropriate drugs among 564 used drugs were assessed according to the criteria of Beers and TIME-to-STOPP, and the most frequently used potentially inappropriate drug was found to be PPI in both criteria [45]. The web application we used in our study gave us the opportunity to assess potentially inappropriate drug use by age according to the criteria of TIME-to-STOPP, AGS Beers 2019, US-FORTA, Stopp/Start v2, EU(7)PIM, and Priscus 1.0 List. The most commonly used potentially inappropriate drug was PPI both in our study and in the literature [43,44,45,46]. While prescribing PPI to patients, other drugs used by the patients should also be evaluated, and it should be prescribed only if it has indications for patients. Inappropriate drug use is not only harmful to the health of patients but also increases the costs of health expenses, which appears a crucial problem. It is possible to say that starting web-assisted applications for rational drug use to prevent patients from inappropriate drug use in busy polyclinic conditions will be beneficial.

On the other hand, the desired results in the struggle against polypharmacy may not be generally achieved as the need for additional time to detect inappropriate drug use cannot be met due to patient density, and several drug interactions may not be considered due to the lack of knowledge or due to the fact that guide evaluation is performed or remembered in daily practice. As a result of the cost-effectiveness analysis, our web assistant, which we presented as the gold standard method in other studies by us and which was revealed as an alternative method with several detected advantages (2 times more inclusive and 60 times faster than the largest PIM criterion [7]) compared to the traditional manual methods, was revealed as a more cost-effective method compared with the alternative “doing nothing” used in cost-effectiveness analyses. Previous states of the related patient groups before they were registered in our training family health center support this detection. Likewise, although inappropriate drug use was presented by traditional methods in many health services, several inappropriate drug uses that could not be detected or interfered with were observed, as revealed in our study.

There was a positive correlation between the number of drugs involved in drug–drug interactions, the number of drugs involved in inappropriate drug use above the age of 65, total number of diseases, and the number of drugs used. In other words, as inappropriate drug use above the age of 65, the total number of diseases, and the number of drugs used increased, the number of drugs involved in drug–drug interactions increased in our study.

There was no significant correlation between the number of drugs detected as red risk in the web application and the total number of diseases; however, there was a positive significant correlation with the number of drugs used. Drugs detected as red risk represented those with contraindications. According to that result, it was not the number of diseases but the number of drugs used that increased the risk of use of drugs with contraindications in patients. In this regard, the importance of searching the interactions between the drugs used while regulating the treatments of patients comes out. It is obvious that the rational drug use web assistant we used easily met this need.

While there was no significant correlation between the drug costs planned to be ceased with the web application, the number of diseases, the number of drugs used, and the number of drugs involved in drug–drug interactions, there was a positive significant correlation with the number of drugs involved in inappropriate drug use above the age of 65. It is evident that inappropriate drug use above the age of 65 increases drug costs here, which comes out as an important issue. It can be seen that, when reflected in the total geriatric population in Turkey, this additional cost would make a huge amount. While such a high amount appears when only the direct costs of drugs are considered, it is an unforgettable fact that these numbers will increase more when we consider additional health problems caused by the potential side-effects of these drugs and hospital stays, even intensive care stays depending on these problems. In addition to all of these, the condition may become more nonignorable as it will cause loss of labor and inefficient use of limited resources. Therefore, a rational drug use web assistant is needed to decrease inappropriate drug use by rapidly detecting inappropriate drug use above the age of 65 even for the decrease of these costs only, and it has been well understood once again that the use of this web assistant by all healthcare systems is a necessity.

### Limitations of the Study

Inappropriateness of some drug agents in advanced age depends on the dose and exposure time of the drug agent. For example, use of PPIs for more than 8 weeks is inappropriate according to the inappropriateness criteria of most drugs. In our study, statistical analysis was performed assuming that patients in our sample had been using their drugs for a long time, which is among the limitations of our study. Additionally, the number of nursing home patients in our study is not sufficient to ensure broad generalizations for the broader population. Nevertheless, our web-based assistant holds the potential to become a pivotal tool for optimizing patient treatments. Follow-ups of the related population must be performed in the long term in order to reveal the health outcomes more clearly; however, the health outcomes in our study could not be assessed with cost-effectiveness analyses such as QALY (Quality Adjusted Life Year) and DALY (Disability Adjusted Life Years), which is another limitation of our study. Instead of that, costs of the drugs and polypharmacy outcomes were used in cost-effectiveness analyses in our study.

## 5. Conclusions

When drug use of patients in nursing homes was assessed with a rational drug use web assistant in our study it was concluded that most of the patients had polypharmacy and potential drug–drug interaction. In addition, there was inappropriate drug use in most of the patients above the age of 65, and it was detected that this increased drug costs, which appears a crucial problem. A significant decline in terms of polypharmacy was observed with the regulation after the related recommendations of the web assistant and the physician’s decision. Therefore, about one-third of patients in whom polypharmacy was detected were kept from several potential side-effects and costs (direct, indirect, or intangible) of polypharmacy. It was concluded in our study that the discontinuation of drugs due to inappropriate drug use in a group including about 100 individuals could directly decrease drug costs by a rate of 10%. According to this, it was observed that the web assistant was more cost-effective than the traditional manual calculation method. Moreover, the total number of active agents used by all patients revealed that no contraindicated drug–drug interaction remained after the number of active agents was decreased by a rate of 12.15% (*n*: 83) following the web assistant and the physician’s decision.

In this specific context, the web application, supported by artificial intelligence and developed by researchers with the primary goal of promoting rational drug utilization, has been recognized for its ability to offer substantial convenience to physicians.

In addition, it has been well understood once again in this study that significant responsibilities fall upon family physicians who can deal with all health problems and medications of patients, approach them in a holistic way, provide care coordination, and base on quaternary prevention.

Moreover, it is considered that this study will raise awareness about the rational drug use web assistant to physicians, guide large masses by encouraging the use of this application in their clinics, and contribute to the proliferation of its national and international use for its ease of application.

## Figures and Tables

**Table 1:** Sociodemographic information and disease status of patients.

Descriptive Variables (*n* = 99)		n	%
Gender	Female	63	63.6
	Male	36	36.4
Marital status	Widowed/Divorced	47	47.5
	Single	40	40.4
	Married	12	12.2
Educational status	Illiterate	25	25.3
	Primary school	38	38.3
	High school	10	10.1
	University/College	26	26.3
Occupations	Unemployed	44	44.4
	Employee	29	29.3
	Officer	26	26.3
Groups of diseases that patients have * * The ratio among all patients is given.	Cardiovascular diseases	197	31.2
	Psychiatric diseases	105	16.6
	Neurologic diseases	104	16.5
	Endocrinological diseases	46	7.3
	Musculoskeletal diseases	39	6.2
	Urologic diseases	34	5.4
	Chest diseases	30	4.8
	Eye diseases	27	4.3
	Gastrointestinal diseases	16	2.5
Diseases that patients have * * There was more than one disease in patients.	Hypertension	77	77.7
	Depression	44	44.4
	Dementia	44	44.4
	Anxiety	36	36.4
	Coronary artery disease	31	31.3
	Diabetes Mellitus	29	29.3
	Hyperlipidemia	27	27.3
	Osteoporosis	26	26.3
	Protein energy malnutrition	22	22.2

**Table 2:** Number of patients’ drugs, total cost of drugs, number of drugs involved in inappropriate drug use above the age of 65, and number of drugs detected as red risk in web application per patient pre- and post-assessment via the “Artificial Intelligence-Supported Rational Drug Use Web Assistant”.

	*n*	Mean ± Standard Deviation (Per Patient)	*p*
Total number of drugs (pre-assessment)	98	6.97 ± 2.85	*p* < 0.001 *
Total number of drugs (post-assessment)	98	6.12 ± 2.82	
Total cost of drugs per patient (pre-assessment)	98	1261.03 ± 981.76	*p* < 0.001 *
Total cost of drugs per patient (post-assessment)	98	1163.96 ± 940.32	
Number of drugs in inappropriate drug use (pre-assessment)	97 **	2.32 ± 1.58	*p* < 0.001 *
Number of drugs in inappropriate drug use (post-assessment)	97 **	1.50 ± 1.28	
Number of drugs in red risk (pre-assessment)	98	0.49 ± 1.02	*p* < 0.001 *
Number of drugs in red risk (post-assessment)	98	0	

* One sample *t* test. ** One patient is under 65, and the other has no disease.

**Table 3:** Correlation between pre- and post-assessment medication use via the “Artificial intelligence-supported rational drug use web assistant”, patient disease count, and planned drug discontinuation costs.

		1	2	3	4	5	6	7	8
Total Number of Diseases of Each Participant	r	1							
	*p*								
	*n*	99							
2. Initial Number of Drugs Used before Web Application	r	0.653 **	1						
	*p*	<0.001							
	*n*	98	98						
3. Number of Drugs Involved in Drug–Drug Interaction	r	0.494 **	0.914 **	1					
	*p*	<0.001	<0.001						
	*n*	90	90	90					
4. Number of Drugs Involved in Inappropriate Drug Use above the Age of 65	r	0.306 **	0.523 **	0.577 **	1				
	*p*	0.004	<0.001	<0.001					
	*n*	86	86	83	86				
5. Number of Drugs Detected as Orange Risk in Web Application	r	0.436 **	0.855 **	0.908 **	0.418 **	1			
	*p*	<0.001	<0.001	<0.001	<0.001				
	*n*	91	91	90	84	91			
6. Number of Drugs Detected as Red Risk in Web Application	r	0.203	0.234 *	0.306 **	0.408 **	−0.120	1		
	*p*	0.055	0.026	0.003	<0.001	0.259			
	*n*	90	90	90	83	90	90		
7. Last Number of Drugs Planned after Web Application	r	0.625 **	0.953 **	0.832 **	0.353 **	0.859 **	0.031	1	
	*p*	<0.001	<0.001	<0.001	0.001	<0.001	0.773		
	*n*	98	98	90	86	91	90	98	
8. Cost of Drugs Planned to be Discontinued with Web Application	r	0.138	0.168	0.149	0.408 **	−0.181	0.788 **	−0.088	1
	*p*	0.174	0.098	0.160	<0.001	0.085	<0.001	0.390	
	*n*	99	98	90	86	91	90	98	99

* Correlation is significant at the level of 0.05 (2-tailed). ** Correlation is significant at the level of 0.01 (2-tailed).

## Data Availability

The data presented in this study are available upon request from the corresponding author. The data are not publicly available due to ethical and legal issues.
